# Gender Differences Are a Leading Factor in 5-Year Survival of Patients with Idiopathic Pulmonary Fibrosis over Antifibrotic Therapy Reduction

**DOI:** 10.3390/life15010106

**Published:** 2025-01-16

**Authors:** Pasquale Tondo, Giulia Scioscia, Cosimo C. De Pace, Fabiola Murgolo, Federica Maci, Giulia M. Stella, Dalila Pescatore, Maria Pia Foschino Barbaro, Donato Lacedonia

**Affiliations:** 1 Department of Medical and Surgical Sciences, University of Foggia, 71122 Foggia, Italy; pasquale.tondo@unifg.it (P.T.); giulia.scioscia@unifg.it (G.S.); fabiolamurgolo3@gmail.com (F.M.); alilad.pes@gmail.com (D.P.); mariapia.foschino@unifg.it (M.P.F.B.); 2 Pulmonary and Critical Care Unit, Department of Specialistic Medicine, University-Hospital Polyclinic of Foggia, 71122 Foggia, Italy; 3 Respiratory Diseases and Rehabilitation Unit, “Teresa Maselli Mascia” Hospital, 71016 San Severo, Italy; cosimo.de.pace26@gmail.com; 4 Pulmonology Unit, “A. Perrino” Hospital, 72100 Brindisi, Italy; fede.maci91@gmail.com; 5 Department of Internal Medicine and Medical Therapeutics, University of Pavia, 27100 Pavia, Italy; g.stella@smatteo.pv.it; 6 Respiratory Diseases Unit, Cardiothoracic and Vascular Department, IRCCS San Matteo Polyclinic Hospital, 27100 Pavia, Italy

**Keywords:** IPF, survival, nintendanib, pirfenidone, antifibrotic treatment

## Abstract

Background: Idiopathic pulmonary fibrosis (IPF) is a chronic, progressive lung disease with a median survival of 3–5 years. Antifibrotic therapies like pirfenidone and nintedanib slow progression, but the outcomes vary. Gender may influence disease presentation, progression, and response to treatment. This study evaluates the impact of gender on the 5-year survival, pharmacological management, and clinical outcomes of patients with IPF. Methods: A retrospective cohort study of 254 IPF patients was conducted, with 164 (131 males:33 females) having complete data. Patients underwent spirometry, DLCO, and 6 min walk tests. Data on comorbidities, smoking, antifibrotic therapy type, dosage adjustments, and adverse events were collected. We used Kaplan–Meier survival curves and logistic regression to assess gender-related differences in outcomes. Results: Men had worse lung function at diagnosis (FVC 74.9 ± 18.5 vs. 87.2 ± 20.1% of pred.; *p* < 0.001) and a higher smoking prevalence (74% vs. 30%; *p* < 0.001). Women had better survival (51.2 vs. 40.8 ± 19.2 months; *p* = 0.005) despite more frequent biopsy use (36% vs. 17%; *p* = 0.013). Women tolerated longer therapy better (*p* = 0.001). No differences were found between patients receiving reduced antifibrotic dosing and those receiving full dosing. Conclusions: Gender has a significant impact on IPF outcomes, with women demonstrating better survival and tolerance to long-term therapy. In contrast, reducing antifibrotic treatment does not appear to significantly affect survival outcomes. These findings underscore the need for future research on gender-specific management approaches.

## 1. Introduction

Idiopathic pulmonary fibrosis (IPF) is the most common and severe disease among idiopathic interstitial pneumonias. It is a progressive interstitial lung disease of unknown etiology that affects 3 million people worldwide. Although its clinical course is extremely variable, the prognosis remains generally bleak, with a median survival of only 3–5 years from diagnosis [1,2].

Despite the significant morbidity and mortality associated with this disease, the lack of specific national registries and the complexity in diagnosing and monitoring the disease make it challenging to obtain precise epidemiological data. Recent insights into the molecular mechanisms of pulmonary fibrosis have highlighted the role of acute lung inflammation in driving fibrotic processes. These findings, supported by in vivo models, offer new insights into both prognosis and therapeutic strategies [3]. Some studies have shown a predominance of the disease in males, who represent 60–70% of total cases, with diagnoses typically occurring between the fifth and seventh decades of life [4].

Several investigations have been conducted on gender differences in the onset and progression of the disease. A study conducted in France [5] highlighted that women tend to present with less advanced disease at diagnosis, better lung function, and more preserved forced vital capacity (FVC). This is possibly due to lower exposure to triggering agents such as tobacco smoke or occupational inhalants compared to men, although future changes in lifestyle habits may influence this.

Additionally, it has been demonstrated that honeycombing and emphysema are more typical in men. This finding is consistent with a previous study that suggested a higher rate of lung biopsy diagnoses in women with IPF [6]. The disease tends to present at an older age in women, and while the progression and overall survival remain comparable regardless of gender, even less use is made of lung transplantation in women. A study by Han MK et al. showed that males with IPF presented more frequently with latent respiratory failure on exertion than females and that survival was better in females [7]. However, although gender may enhance the diagnostic reliability, the association between gender and disease must be supported by validated data to avoid inaccurate diagnoses or underdiagnoses [8]. Thus, gender can influence diagnosis, with a tendency to overdiagnose IPF in men and underdiagnose it in women. Additionally, gender may also affect disease management and therapy. In a study conducted in Sweden, the comorbidities that were most commonly found in the two sexes were highlighted; it was found that in males, coronary artery disease and other cardiovascular diseases, such as atrial fibrillation and heart failure, predominate. On the other hand, in women, especially non-smokers, the most commonly observed comorbidities are gastroesophageal reflux disease and asthma [6]. Most interestingly, GERD has been increasingly recognized not only as a frequent comorbidity in IPF but also as a potential factor in the disease progression. A debate remains whether GERD should be actively treated in these patients to mitigate its impact on the clinical outcomes [9].

The goals of IPF management are to ameliorate symptoms, improve health status, preserve lung function, maintain adequate oxygenation with supplemental oxygen when needed, minimize adverse events of therapy, reduce the frequency of acute exacerbations, and, ideally, improve survival [10].

Unfortunately, there is no curative treatment other than lung transplantation. Currently, two antifibrotic therapies have been approved for IPF: pirfenidone and nintedanib. Both drugs have been associated with significantly slowing disease progression and respiratory deterioration in IPF and potentially prolonging survival.

Recent studies have further supported these findings. A systematic review comparing pirfenidone and nintedanib confirmed their efficacy in slowing disease progression while highlighting differences in their tolerability and side effect profiles, which may guide clinical decision making [11]. Preclinical research has shown that both pirfenidone and nintedanib attenuate lung fibrosis in mice by inhibiting JAK2, a critical mediator in fibrotic pathways. This inhibition leads to reduced expression of the TGF-β receptor and smooth muscle actin α, a marker of myofibroblast activation, which are central in fibrogenesis [12].

Furthermore, although both therapies are effective, their different strengths and limitations underscore the importance of individualized treatment approaches. A promising future strategy could involve combining pirfenidone and nintedanib, as their complementary mechanisms of action target different aspects of fibrogenesis. This synergy could increase the therapeutic efficacy compared with monotherapy. However, the main concern with combination therapy is the potential increase in adverse events. Recent studies indicate that these side effects are manageable with appropriate clinical strategies, supporting the feasibility of this approach [13].

However, the response to antifibrotic treatment is heterogeneous and may be limited by side effects, demonstrating the constant need to establish novel therapeutic approaches, including combination therapies and the development of new compounds [14,15].

We hypothesize that changes in therapy, specifically reductions, may impact the outcomes of patients with IPF differently. Therefore, the purpose of this study is to evaluate the survival outcomes of patients undergoing antifibrotic therapy and survival after therapy adjustment and, particularly, to assess the presence of gender differences in both the general characteristics and therapeutic management and survival of patients with IPF.

## 2. Materials and Methods

### 2.1. Study Design and Diagnostic Criteria

This is a single-center retrospective cohort study. Data were retrospectively collected from a cohort of 254 patients who were diagnosed with IPF and admitted to our unit from October 2011 to July 2023.

All patients underwent a series of diagnostic tests, including spirometry, diffusing capacity of the lung for carbon monoxide (DL_CO_), and the 6 min walk test (6MWT). Survival data for patients who were included in the study were collected at 60 months from diagnosis. The diagnosis of IPF was made through an in-depth discussion by a multidisciplinary team, in accordance with the international guidelines.

To establish the diagnosis of IPF, both clinical and radiological data were considered. The diagnosis was confirmed in the presence of a typical pattern on high-resolution computed tomography (HRCT), known as usual interstitial pneumonia (UIP), or a “probable UIP” pattern in an appropriate clinical context. In cases where the typical pattern on HRCT could not be confirmed, the diagnosis of IPF was based on a combination of compatible clinical data and, when necessary, lung biopsy.

### 2.2. Pharmacological Protocol

At IPF diagnosis, patients started antifibrotic treatment (pirfenidone or nintedanib) according to the criteria of the European Medicines Agency (EMA), Food and Drug Administration (FDA), and Italian Medicines Agency (AIFA).

nintedanib:

- Inclusion Criteria: Age ≥ 40 years, IPF diagnosis according to international guidelines, FVC > 50% of predicted, and DL_CO_ > 30%.
- Exclusion Criteria: ALT and/or AST > 1.5× ULN, total bilirubin >1.5× ULN, high risk of bleeding, INR > 2, PT/PTT > 150% of ULN, scheduled major surgery in the next 3 months, and high risk of thrombosis.

The recommended dose is 150 mg twice daily with food, approximately 12 h apart. For patients who cannot tolerate this dose, it can be reduced to 100 mg twice daily. If symptoms do not resolve, the treatment should be temporarily discontinued and then resumed at either the full dose (150 mg twice daily) or the reduced dose (100 mg twice daily). If symptoms persist, the medication must be discontinued. Regular blood tests to monitor liver function are required, particularly to check for increases in transaminases (AST or ALT > 3 times ULN), which may necessitate dose reduction or discontinuation. Common side effects include diarrhea, vomiting, nausea, loss of appetite, and abdominal pain.

pirfenidone:

- Inclusion Criteria: Age 40–80 years, IPF diagnosed in the past 48 months, FVC ≥ 50% of predicted value, and DL_CO_ ≥ 35% of predicted value.
- Exclusion Criteria: Hypersensitivity to the active substance or any excipients, severe liver function or terminal liver disease, severe renal impairment (Creatinine Clearance < 30 mL/min), or terminal kidney disease requiring dialysis.

The recommended maintenance dose is 801 mg three times daily, taken with food. Treatment starts at 267 mg three times daily, gradually increasing to the recommended dose of 2403 mg/day over 14 days. Before initiating treatment, blood tests should be performed within the first 15 days and then monthly for the first six months. These tests include complete blood count, transaminases, gamma-glutamyl transferase (GGT), alkaline phosphatase, bilirubin, and creatinine to assess for potential increases in transaminases, which may require dose adjustment or temporary discontinuation. Common side effects include gastrointestinal events, photosensitivity reactions or rashes, and liver function alterations.

### 2.3. Pulmonary Function Assessments

Patients performed pulmonary function tests (PFTs) and DL_CO_ measurements using a calibrated spirometer (Sensormedics, Milan, Italy) according to the ATS/ERS standards [16]. The forced expiratory volume in 1 s (FEV_1_), forced vital capacity (FVC), FEV_1_/FVC ratio, and DL_CO_ corrected for hemoglobin concentration were recorded. To express the PFT results as percentages of the predicted values (% of pred.), we adopted the Global Lung Function Initiative (GLI) reference values throughout the study’s time points [17,18]. After conducting three maneuvers, the best trial in terms of volume, expressed in liters and as a percentage of the predicted normal value, was selected. The DL_CO_ measurement was performed using the single-breath hold maneuver according to guidelines. The average value of two attempts with a variation of less than 10% between them was recorded as the DL_CO_ [19].

To evaluate the functional limitation status and monitor disease progression, patients also performed the 6MWT according to the ATS guidelines [20]. Briefly, study participants were instructed to walk as far as possible for 6 min [21]. The 6MWT was conducted indoors, on a flat, straight corridor that was 30 m long and marked meter by meter. Before starting the test and at the end of its execution, data on SpO_2_, heart rate (HR), and blood pressure were collected. Additionally, upon completing the 6MWT, the distance covered was calculated and recorded.

### 2.4. Statistical Analysis

Data were analyzed using SPSS (IBM Corp., Chicago, IL, USA, version 26). The sample distribution was analyzed using the Shapiro–Wilk test. Continuous data were expressed as mean ± standard deviation, while categorical data were expressed as number and percentage. Based on the sample distribution, a T-test and Wilcoxon test were used for the comparison of continuous variables, and Chi-square was used for categorical variables. Mortality risk factors were searched for with an estimated odds ratio (OR) (95% confidence interval [95% CI]) by a logistic regression test among the variables of sex, smoking history, and comorbidities. A univariate analysis of risk factors was subsequently tested by multivariate analysis. In addition, differences were sought between the two sexes, between the use of one antifibrotic drug versus another, and between antifibrotic drugs dosages, i.e., full or reduced as specified above. Kaplan–Meier survival curves were constructed for these groups and analyzed by the Cox–Mantel test. A *p*-value of less than 0.05 was considered statistically significant.

## 3. Results

### 3.1. Population and Clinical Characteristics

A total of 164 patients with complete outcomes were analyzed: 131 males (80% of the total) and 33 females (20%). The general characteristics of the population and the differences between the two sexes are summarized in Table 1.

The analysis revealed several significant differences between the sexes. Women had better pulmonary function at diagnosis, with higher FEV_1_ (94.8 ± 23.5% vs. 79.4 ± 17.5%; *p* < 0.001) and FVC (87.2 ± 20.1% vs. 74.9 ± 18.5%; *p* = 0.001). In contrast, men were more likely to be smokers (74% vs. 30%; *p* < 0.001) and had higher rates of radiological diagnoses without biopsy (83% vs. 64%; *p* = 0.013). It was noted that 33 patients (20%) had a familial form of IPF: 26 males and 7 females (*p* = 0.862).

Significant differences were also observed in comorbidities: gastroesophageal reflux disease (GERD) was more prevalent in women (48% vs. 13%; *p* < 0.001). No differences were found regarding age at diagnosis (69.3 ± 7.4 vs. 68.5 ± 9.2 years; *p* = 0.626), occupational exposure (21% vs. 22%; *p* = 0.909), or DL_CO_ values (*p* = 0.514). The distance and oxygen saturation of the 6MWT also did not differ.

**Figure 1:**
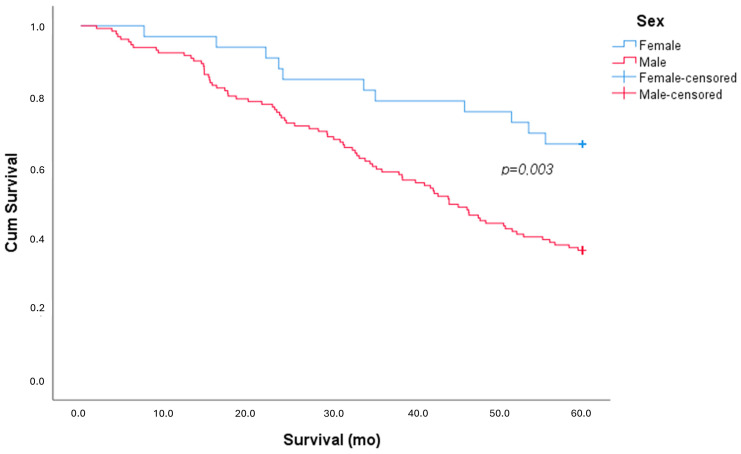
Kaplan–Meier survival curve, stratified by sex.

During the follow-up period, out of 164 patients, we recorded the death of 94 patients (57% of the sample), of which 83 were males (63%) and 11 were females (33%), with a statistically significant difference (X^2^ *p* = 0.002; Cox–Mantel *p* = 0.003), as shown in Figure 1.

The survival analysis showed that women had significantly better outcomes, with a mean survival of 51.2 ± 15.6 months compared to 40.8 ± 19.2 months in men (*p* = 0.005).

When examining mortality risk factors, a univariate analysis highlighted the negative impact of smoking habits (OR 2.39 [95% CI 1.24–4.62]; *p* = 0.009) and male sex (OR 3.36 [95% CI 1.5–7.5]; *p* = 0.003). The multivariate analysis confirmed that male sex (OR 2.65 [95% CI 1.12–6.25]; *p* = 0.026) was a stronger risk factor than smoking (OR 1.8 [95% CI 0.88–3.66]; *p* = NS).

### 3.2. Pharmacological Therapy and Dose Adjustment

All patients were on antifibrotic medication: 73 (45%) were treated with nintedanib—56 (43%) males and 17 (48%) females—while 91 (55%) were treated with pirfenidone, of which 75 (57%) were males and 16 (48%) were females.

Additionally, it was found that 7 (5%) males and 4 (12%) females discontinued therapy (*p* = 0.166), either by voluntary decision or due to adverse effects. The therapy duration was significantly longer in women (51.7 ± 14.6 vs. 39.4 ± 19.2 months; *p* = 0.001).

Mild-to-moderate adverse events occurred in 38 (23%) subjects: 31 (24%) males and 7 (21%) females (*p* = 0.767). Specifically, adverse events included increased liver enzymes, diarrhea, nausea, weight loss, poor appetite, and photosensitivity. No drug-related fatal events were reported.

Overall, 37 patients reduced their antifibrotic therapy (28 males and 9 females), as shown in Table 2.

**Figure 2:**
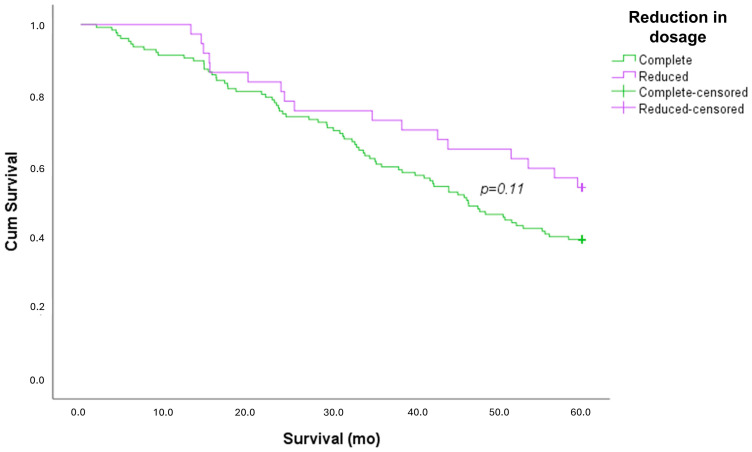
Kaplan–Meier survival curve, stratified by dosage adjustment.

No difference between patients who were treated with a full dosage and reduced dosage was found in terms of the type of diagnosis, risk factors, and comorbidities. More patients who were treated with nintedanib (38% vs. 10% treated with pirfenidone) reduced their therapy (*p* < 0.001). No differences were found in PFTs and 6MWT. A survival curve analysis showed no differences between patients who took a reduced dosage of antifibrotic drugs compared to those who took the full dosage (Figure 2).

## 4. Discussion

The present study evaluated the survival of IPF patients under antifibrotic therapy, stratifying the data by gender, type of medication, and dosage adjustments. Unlike previous studies, which have focused primarily on gender differences in disease prevalence and outcomes, this investigation provides new insights by exploring how antifibrotic therapy is affected by gender, both in terms of survival and therapeutic management. This approach allows for a more comprehensive understanding of therapeutic management and its impact on survival.

One of the primary findings from our investigation is that idiopathic pulmonary fibrosis predominantly affects men; in a sample of 164 patients, 131 were male and 33 were female. This aligns with the literature, as demonstrated by a study conducted in Bernalillo County, New Mexico, which reported a higher incidence of IPF in men than in women. According to this study, IPF affects men with an estimated incidence of 10.7 cases per 100,000 per year, while for women, the incidence is 7.4 cases per 100,000 per year [1].

This clearly indicates that being male is a risk factor for developing IPF, and when considering males over the age of 65, the risk of development and the mortality rate increase significantly [22]. It is interesting to note that women have a higher age of incidence compared to men, particularly over 65 years, as for various biological reasons, IPF may manifest later in women. Gender differences in respiratory pathology begin as early as intrauterine development, when the female airways grow in proportion to lung volume, allowing for higher airflows. During childhood and adolescence, women benefit from a functional respiratory advantage due to larger airways, whereas in men, who develop larger lungs and larger airways, the advantage is reversed in adulthood. Estrogens play a protective role and may delay the progression of IPF in women, contributing to a later onset of symptoms. In summary, the combination of anatomical and hormonal factors could explain why women manifest IPF later than men, who, despite having greater respiratory reserve, are exposed to risk factors earlier, leading to an earlier onset of the disease [23]. Despite this, our study did not find a significant difference in the average age at diagnosis between the two sexes (*p* = 0.626).

Regarding exposure to risk factors, our study found that male smokers had a higher prevalence compared to females (*p* < 0.001), which is consistent with the literature. A recent French study highlighted significant differences between men and women regarding smoking habits and occupational exposure. The study involved 51 female and 185 male IPF patients and found that there was a lower percentage of female smokers than male smokers, with a very high statistical significance (*p* < 0.001). Additionally, women experienced a lower frequency of occupational exposure compared to men. However, in the future, we may expect an increase in respiratory diseases among women due to the growing prevalence of smoking and increased occupational risks [5].

In our study, we evaluated the most common comorbidities in both sexes. A notable finding was the higher incidence of gastroesophageal reflux disease (GERD) in female patients. Although several studies have examined the association between GERD and IPF, few have specifically analyzed gender differences in this relationship [24,25,26]. In particular, a study by Lee et al. examined the association between GERD therapy and survival in IPF patients. Patients who were treated with proton pump inhibitors showed greater survival compared to untreated patients [27]. These findings suggest that GERD may play a role in the progression of IPF, and that effective management of GERD could positively influence the clinical outcomes in IPF patients.

The gender differences observed in IPF are also reflected in other ILDs and respiratory diseases. For example, in hypersensitivity pneumonitis (HP), the etiology and prevalence vary between the sexes: in the United States, women account for 58 percent of cases, while in Denmark, a male predominance (57 percent) is observed. These differences could be related to different exposures to etiologic antigens, such as agricultural or metalworking fluids, to which men are more frequently exposed. In connective tissue-disease-associated ILDs (CTD-ILDs), women are more frequently affected, with ratios as high as 10:1 compared to men for conditions such as lupus erythematosus and systemic sclerosis. However, in the case of rheumatoid arthritis, men more often show a UIP pattern, while women frequently show non-UIP patterns. Significant differences also emerge in asthma and COPD: women with asthma show greater disease severity, while in COPD, women develop more symptomatic forms despite less cumulative smoke exposure. These observations reinforce the importance of considering gender in the diagnosis and management of ILDs and other respiratory diseases [28].

Consistent with the scientific literature, our investigation found that women had a greater need for biopsy to confirm the diagnosis compared to men (*p* = 0.013). Consequently, in most male cases, the approach combining radiological pattern analysis with thorough clinical evaluation and multidisciplinary assessment proved effective in achieving a definitive diagnosis. The diagnosis of IPF primarily relies on HRCT for identifying characteristic radiological patterns. The current literature indicates that defined or probable UIP patterns are more commonly found in male patients, facilitating an IPF diagnosis. Conversely, atypical radiological patterns are more frequent in female patients, often necessitating surgical lung biopsy to confirm the diagnosis of IPF [8].

Furthermore, the therapy tolerance and dosage adjustments differ between men and women. After five years of antifibrotic treatment (nintedanib vs. pirfenidone), about one-third of patients had to reduce their nintedanib dosage, while only 10% of patients who were treated with pirfenidone did. Overall, the discontinuation rate (permanent or temporary) is low for both drugs.

Patients on reduced-dose treatment showed a similar survival rate to those on full-dose treatment. This evidence shows the efficacy of antifibrotic therapy, despite the reduction in dosage.

These results are in line with previous studies. For example, a prospective post-marketing study of pirfenidone conducted in 10 hospitals in South Korea from 2014 to 2017 confirmed that reduced doses did not negatively impact the clinical outcomes compared to standard-dose pirfenidone in IPF patients [29]. Dose reduction may be a useful method to manage adverse events while maintaining the therapeutic efficacy. Porse et al., in their article, analyzed a Danish population of IPF patients from April 2016 to November 2021, confirming this hypothesis for both antifibrotic drugs. In their cohort, nearly 80% of patients received reduced doses of nintedanib and 67% of pirfenidone, with no significant worsening in survival outcomes compared to full-dose-treated patients [30].

Therefore, the implementation of a targeted therapeutic approach that takes individual differences in drug tolerance and response into account could help reduce the need for invasive interventions, such as lung transplantation, and significantly improve the quality of life of IPF patients. This becomes particularly relevant considering recent studies focusing on the identification of biomarkers that are predictive of the response to antifibrotic treatments. Biomarkers such as TERT, TERC, SP-D, periostin, TGF-β, IL-6, and KL-6 have emerged as promising indicators to provide crucial information regarding the disease progression, drug tolerance, and patient prognosis [31,32,33]. In particular, KL-6 has been shown to be a significant indicator of the disease severity and response to treatment, suggesting that monitoring of this biomarker could more accurately guide therapeutic decisions [32,34].

This study also confirms that women with IPF have a better prognosis, as was known in the literature, so much so that in the GAP index, being female has a smaller impact on the prognosis, almost as if the disease is genetically different between men and women. However, no gender differences emerged in terms of reduction in/discontinuation of therapy, nor did this impact mortality. Furthermore, in our recent study, we observed that the real-life mortality in patients who were under antifibrotic treatment was similar to the untreated population based on which the GAP index [35] was first proposed. It is unethical to plan a new pharmacological comparison protocol between treated and untreated patients with the current knowledge and diagnosis time; however, as already cited, this result leaves a veil of uncertainty that should be investigated in further analyses.

All these considerations should open a new debate on the usefulness of antifibrotic therapy or, at least, on the usefulness of the full dosage with the increased adverse effects in fragile patients.

This study has limitations, mainly due to its retrospective nature; patients were not randomized, and therefore, there are no uniform groups with similar baseline characteristics; additionally, the entire analyzed population is pharmacologically treated, and comparison with a placebo group is not possible; pre-treatment respiratory function parameters were only available for a few patients, which is why it was not possible to assess the difference in functional decline between the pre-treatment and post-treatment periods.

## 5. Conclusions and Future Perspectives

The results of our study suggest that IPF has distinct characteristics for men and women, highlighting the importance of gender differences in disease pathogenesis and treatment tolerance. Furthermore, our findings challenge the general belief in the efficacy of full therapeutic dosing by suggesting a similar efficacy of reduced treatment. With this statement, we do not aim to discredit the importance of antifibrotic treatment compliance but to stimulate further research that identifies factors relating to these findings.

It is critical that future research will include more detailed gender-specific analyses to re-fine therapeutic strategies and improve the management of IPF. To this end, it is essential to promote clinical trials that disaggregate data by gender, support research on gender-specific drug treatments, and identify biomarkers that predict susceptibility and response to therapies. These strategies must take the different clinical presentations, biological mechanisms, and pharmacokinetic profiles that are observed between men and women into account, addressing heterogeneity in the disease manifestation and response to treatment.

In addition, it is important to periodically update national guidelines to include gender-specific indicators and implement targeted training programs for health care providers. These programs should include courses and seminars that are aimed at increasing the awareness of gender biases and their potential impacts on clinical decision making.

Finally, identifying environmental risk factors, both internal and external, that may affect men and women differently is another key area for future research. Targeted interventions in these areas will help optimize the management of IPF, improving therapeutic outcomes and ensuring more equitable care for all patients.

## Figures and Tables

**Table 1:** Study population characteristics and comparison between sexes.

	Total	Male	Female	*p*
	*N* = 164	*N* = 131	*N* = 33	
Age at diagnosis (year)	69.1 ± 7.7	69.3 ± 7.4	68.5 ± 9.2	0.626
Sex (male)	131 (80%)			
Type of diagnosis				
Histological diagnosis	34 (21%)	22 (17%)	12 (36%)	0.013
Radiological diagnosis	130 (79%)	109 (83%)	21 (64%)	0.013
Risk factors				
Smoking habits	107 (65%)	97 (74%)	10 (30%)	<0.001
Packs per years	44.3 ± 30.9	45.8 ± 31.0	23.9 ± 21.2	0.127
IPF familiar	33 (20%)	26 (20%)	7 (21%)	0.862
Occupational exposure	36 (22%)	29 (22%)	7 (21%)	0.909
Comorbidities				
GERD	33 (20%)	17 (13%)	16 (48%)	<0.001
Pulmonary hypertension	39 (24%)	34 (26%)	5 (15%)	0.195
Pulmonary neoplasm	3 (2%)	3 (2%)	0 (0%)	0.383
Depression	8 (5%)	5 (4%)	3 (9%)	0.211
CVD	95 (58%)	77 (59%)	18 (55%)	0.662
Rheumatological diseases	17 (10%)	16 (12%)	1 (3%)	0.123
Antifibrotic therapy				
nintedanib	73 (45%)	56 (43%)	17 (52%)	0.368
pirfenidone	91 (55%)	75 (57%)	16 (48%)	0.368
Suspension therapy	11 (7%)	7 (5%)	4 (12%)	0.166
Reduction in dosage	37 (23%)	28 (21%)	9 (27%)	0.472
Reduction in dosage nintedanib/pirfenidone	28/9	21/7	7/2	<0.001
Duration of therapy (mo)	41.9 ± 18.9	39.4 ± 19.2	51.7 ± 14.6	0.001
AE *	38 (23%)	31 (24%)	7 (21%)	0.767
Pulmonary performance				
FEV_1_ (L)	2.0 ± 0.6	2.1 ± 0.5	1.6 ± 0.4	<0.001
FEV_1_ (% of pred.)	82.4 ± 19.7	79.4 ± 17.5	94.8 ± 23.5	<0.001
FVC (L)	2.4 ± 0.8	2.6 ± 0.7	1.8 ± 0.5	<0.001
FVC (% of pred.)	77.4 ± 19.4	74.9 ± 18.5	87.2 ± 20.1	0.001
DL_CO_	50.5 ± 14.8	50.1 ± 15.0	52.1 ± 14.1	0.514
6MWT (mt)	352.0 ± 135.8	356.8 ± 136.2	327.1 ± 134.2	0.361
SpO_2_ (6MWT start)	95.7 ± 2.0	95.6 ± 2.1	96.3 ± 1.7	0.171
SpO_2_ (6MWT end)	87.3 ± 10.1	87.4 ± 6.5	86.5 ± 19.8	0.698
Survival				
Exitus	94 (57%)	83 (63%)	11 (33%)	0.002
Survival (mo)	42.9 ± 19.0	40.8 ± 19.2	51.2 ± 15.6	0.005

Continuous data are expressed as mean ± standard deviation, while categorical data are expressed as a number (percentage). Abbreviations: 6MWT = 6 min walking test; AEs = adverse effect events; CVDs = cardiovascular diseases; DL_CO_ = diffusing capacity of the lungs for carbon monoxide; FEV1 = forced expiratory volume in the 1st second; FVC = forced vital capacity; GERD = gastroesophageal reflux disease; IPF = idiopathic pulmonary fibrosis; PH = pulmonary hypertension. * AEs include increased liver enzymes, diarrhea, nausea, weight loss, loss of appetite, and photosensitivity.

**Table 2:** Comparison between patients who were treated with full dosage or reduced dosage of antifibrotic drugs.

	Complete	Reduced	*p*
	*N* = 127	*N* = 37	
Age at diagnosis (year)	69.5 ± 6.9	68.0 ± 10.2	0.31
Sex (male)	103 (81%)	28 (76%)	0.472
Antifibrotic therapy			
nintedanib/pirfenidone	62%/90%	38%/10%	<0.001
Duration of therapy (mo)	40.5 ± 19.2	46.9 ± 17.4	0.07
AE *	26 (20%)	12 (32%)	0.131
Pulmonary performance			
FEV_1_ (L)	2.0 ± 0.6	1.9 ± 0.4	0.292
FEV_1_ (% of pred.)	83.9 ± 19.9	77.3 ± 18.4	0.104
FVC (L)	2.4 ± 0.8	2.3 ± 0.7	0.532
FVC (% of pred.)	78.1 ± 19.4	75.2 ± 19.4	0.423
DL_CO_	50.6 ± 14.4	50.2 ± 16.1	0.896
6MWT (mt)	346.2 ± 140.5	370.4 ± 119.8	0.389
SpO_2_ (6MWT start)	95.6 ± 2.1	96.3 ± 1.6	0.099
SpO_2_ (6MWT end)	87.5 ± 6.6	86.6 ± 17.2	0.699
Survival			
Exitus	77 (61%)	17 (46%)	0.113
Survival (mo)	41.6 ± 19.2	47.3 ± 17.8	0.112

Continuous data are expressed as mean ± standard deviation, while categorical data are expressed as a number (percentage). Abbreviations: 6MWT = 6 min walking test; AEs = adverse effect events; DL_CO_ = diffusing capacity of the lungs for carbon monoxide; FEV1 = forced expiratory volume in the 1st second; FVC = forced vital capacity. * AEs include increased liver enzymes, diarrhea, nausea, weight loss, loss of appetite, and photosensitivity.

## Data Availability

The data presented in this study are available on request from the corresponding author.
